# Chemical Interaction-Induced Evolution of Phase Compatibilization in Blends of Poly(hydroxy ether of bisphenol-A)/Poly(1,4-butylene terephthalate)

**DOI:** 10.3390/ma11091667

**Published:** 2018-09-09

**Authors:** Jing Liu, Hsiang-Ching Wang, Chean-Cheng Su, Cheng-Fu Yang

**Affiliations:** 1 School of Information Engineering, Jimei University, Xiamen 361021, China; jingliu@jmu.edu.cn; 2 Department of Chemical and Materials Engineering, National University of Kaohsiung, No. 700, Kaohsiung University Rd., Nan-Tzu Dist., Kaohsiung 811, Taiwan; celestine772000@yahoo.com.tw

**Keywords:** immiscible blend, compatibilization, homogeneous phase, alcoholysis, copolymers

## Abstract

An immiscible blend of poly(hydroxy ether of bisphenol-A) (phenoxy) and poly(1,4-butylene terephthalate) (PBT) with phase separation was observed in as-blended samples. The compatibilization of phenoxy/PBT blends can be promoted through chemical exchange reactions of phenoxy with PBT upon annealing. The annealed phenoxy/PBT blends had a homogeneous phase with a single *T*_g_ that could be enhanced by annealing at 260 °C. Infrared (IR) spectroscopy demonstrated that phase homogenization could be promoted by annealing the phenoxy/PBT blend, where alcoholytic exchange occurred between the dangling hydroxyl group (–OH) in phenoxy and the carbonyl group (C=O) in PBT in the heated blends. The alcoholysis reaction changed the aromatic linkages to aliphatic linkages in the carbonyl groups, which initially led to the formation of a graft copolymer of phenoxy and PBT with an aliphatic/aliphatic carbonyl link. The progressive alcoholysis reaction resulted in the transformation of the initial homopolymers into block copolymers and finally into random copolymers, which promoted phase compatibilization in blends of phenoxy with PBT. As the amount of copolymers increased upon annealing, the crystallization of PBT was inhibited by alcoholytic exchange in the blends.

## 1. Introduction

Blending immiscible polymers offers attractive opportunities for developing new materials with useful combinations of properties. Compatibilization and phase homogeneity in the blending of immiscible polymers can be enhanced by physical interactions such as van der Waals forces, dipole–dipole interactions and hydrogen bonding, or chemical interactions (reactive compatibilization) such as the formation of covalent bonds between polymers [1,2,3]. In physical blending, preformed graft or block copolymers are traditionally added to act as compatibilizers. Reactive blending is employed to generate these copolymer compatibilizers in situ during melt blending or annealing using functionalized polymers. Generally, reactive blending has several advantages over physical blending, based on the utility and controllability of the polymer processing. In reactive blending, chemical reactions between reactive groups progress during melt blending or heat treatment. Thus, a phase-separated compatibilized blend can be achieved with controllable morphology and interfaces [2,3,4,5,6,7].

Phenoxy contains ether linkages in the backbone and pendant hydroxyl groups that exhibit outstanding mechanical properties, such as toughness and dimensional stability [3,5,8,9]. In its organic chemistry, the oxygen in the hydroxyl group has two lone pairs with negative charges on the para and ortho positions, which become the activated positions when electron-donating substituents are present [10]. The hydroxyl group in the phenoxy molecule is attached to an aliphatic structure, in which the hydroxyl group is electron-withdrawing and thus strongly activating. The hydroxyl group of phenoxy can interact with proton-accepting functional groups in other polymers in polymer blends. The compatibility of phenoxy and other polymers in polymer blends can be increased by both physical (hydrogen bonding) and chemical (alcoholysis reaction) interactions [8,9].

If there is a large difference in the electronegativity of functional groups in the components of the blends, a strong physical interaction (hydrogen bonding) can enhance their compatibility [11]. The compatibility of polymer blends containing phenoxy typically originates from hydrogen bonding between the hydroxyl group of phenoxy and proton-accepting functional groups of the other polymers, such as polyoxides [12], polyamides [13], polyesters [14,15], poly(methyl methacrylate) [16], poly(ε-caprolactone) (PCL) [17], poly(vinylpyrrolidone) [18], and phenol resin [19]. Coleman et al. [17] studied the strong physical interactions in phenoxy/PCL blends using Fourier-transform infrared (FTIR) spectroscopy. The main conclusion was that the hydrogen-bonded hydroxyl infrared (IR) absorption shifted to lower frequencies as the PCL concentration changed. Such blends exhibit significant shifting of the IR absorption of the hydroxyl group, which is ascribed to a distinct interaction between the phenoxy and polymers. This implies that the hydrogen bonding interaction is stronger than the corresponding self-associated hydrogen bonding in neat phenoxy.

Additionally, phenoxy can also form immiscible or partially miscible blends with some aliphatic polyesters (e.g., poly(ethylene succinate) [20], poly(butylene acid) (PBA) [5], and poly(3-hydroxybutyrate) (PHB) [21]) and aromatic polyesters (e.g., poly(ethylene terephthalate) (PET) [22], poly(trimethylene terephthalate) (PTT) [23], and poly(ethylene 2,6-naphthalenedicarboxylate) [24]). However, the chemical interactions that occur following high-temperature annealing enhance miscibility. In the molecular structure of polyesters, the carbonyl group is polar (the electronegativity of oxygen is larger than that of carbon; therefore, the carbonyl group has a large dipole moment); thus, the carbonyl carbon atom is partially positively charged and hence can act as an electrophile. Therefore, these molecules easily undergo nucleophilic substitution reactions [25], so the hydroxyl groups of phenoxy can take part in a specific chemical reaction, that is, alcoholysis, with polyesters. During reactive blending, the interfacial chemical reactions form copolymers in situ, which suppress coalescence and reduce interfacial tension. As a result of this, a stable and fine morphology is attained with enhanced interfacial adhesion between the phases. Additionally, compatibilization can introduce reactive molecules that are capable of forming the desired copolymers, both in situ and directly during the blending or annealing of the reactive polymer blends [2,3,4,5].

Our study aims to contribute to an understanding of how to control the compatibilization of phenoxy and poly(1,4-butylene terephthalate) (PBT). Blends of different compositions were produced and melt-annealed, resulting in the chemical interaction-induced evolution of phase compatibilization in blends of phenoxy with PBT. Analyses were performed to investigate the chemical interactions, the influence of the annealing procedure on the degree of chemical interaction, and the influence of the chemical interactions on the crystallization behavior. Changes in the thermal behavior, morphology, and molecular structures of blends with various compositions were investigated and discussed in relation to the chemical interactions.

## 2. Experimental

### 2.1. Materials and Methods

Phenoxy was purchased from Scientific Polymer Products (New York, NY, USA), with an average molecular weight (M_n_) of 23,000 g·mol^−1^, a weight-average molecular weight (M_w_) of 80,000 g·mol^−1^, and a glass transition temperature (*T*_g_) of 90 °C. PBT was a research-grade resin with no additives, obtained from Chang Chun Corp. (Hsinchu, Taiwan). The aryl polyester PBT is semicrystalline, with a *T*_g_ of 35 °C, a M_n_ of 25,000 g·mol^−1^ and an apparent melting temperature (*T*_m_) of 225 °C. The chemical structures of the repeating units of PBT and phenoxy are as follows:


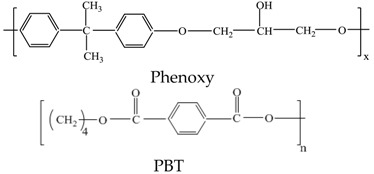


To ensure that the inherent phase behavior of the physical blends was initially understood, blend specimens were prepared by solution blending to avoid thermal heating effects at elevated temperatures, which are inevitable in melt blending. Blends of phenoxy with PBT were prepared by solution blending/casting using hexafluoroisopropanol (HFIP, C_3_H_2_OF_6_) as a good mutual solvent. The two constituent polymers at a concentration of 0.04 g·mL^−1^ in solution were mixed in the desired proportions, stirred thoroughly, and cast onto glass dishes at 50 °C. The solvent was evaporated at 50 °C for 24 h, then samples were degassed for one week in a vacuum oven at 80 °C to remove the residual solvent prior to characterization. Blend compositions of phenoxy/PBT were fixed at 10/90, 30/70, 50/50, 70/30, and 90/10. These samples were designated “as-blended.” As-blended materials were further held at 260 °C for different periods of time (0–180 min) to evaluate the effects of heat annealing. These latter samples were designated “heat-annealed.” The different blend compositions were then exposed to a temperature of 260 °C for various periods of time. The soluble portions of the reacted samples were extracted with HFIP. The residual solids and extracted solution were kept separately for FTIR analysis. Throughout the annealing process, dry nitrogen was continuously purged over the annealing chamber to minimize degradation/oxidation, which decreased the influence of the thermal-induced degradation of the polymeric phase on the morphological evolution in the phenoxy/PBT blends. The thermal stability of the single polymeric phases was examined using thermogravimetric analysis (TGA) (TGA 2950, TA Instruments Inc., New Castle, DE, USA). It was apparent that unobvious mass loss occurred for phenoxy and PBT at 260 °C for 3 h.

### 2.2. Characterization

#### 2.2.1. Differential Scanning Calorimetry

The thermal behavior of the blends, such as *T*_g_, *T*_m_ and nonisothermal crystallization, were characterized using the PerkinElmer DSC-7 differential scanning calorimeter (DSC) (PYRIS I, PerkinElmer Inc., Waltham, MA, USA). *T_g_* values were taken as the onset temperature of the transition of the heat flow curves. Samples were heated rapidly from 0 °C to 260 °C, held at this temperature for various annealing times (*t*_a_), and then cooled at a rate of 10 °C·min^−1^ to 0 °C·min^−1^.

#### 2.2.2. Scanning Electron Microscopy

In order to confirm the phase structure of the polymer mixtures, the morphology of the fracture surfaces of the blends was examined using a scanning electron microscope (SEM) (HF-2000, Hitachi Inc., Tokyo, Japan).

#### 2.2.3. Fourier Transform Infrared (FTIR) Spectroscopy

FTIR spectroscopy (PerkinElmer Spectrum 100, PerkinElmer Inc., Waltham, MA, USA) was used to investigate the molecular interactions between the constituents. Spectra were obtained at a resolution of 4 cm^−1^, and averages were obtained from at least 128 scans in the wavenumber range 400–4000 cm^−1^. Two spectroscopic techniques were used. Thin films for FTIR studies were obtained by casting phenoxy/PBT solutions onto potassium bromide (KBr) disks at 50 °C and then removing them in vacuum at 80 °C. To evaluate the chemical reactions at a high temperature, samples were subjected to appointed thermal treatments (isothermal 260 °C for 3 h). Samples cast on KBr pellets were heated, cooled to ambient temperature, and studied using FTIR spectroscopy. 

## 3. Results and Discussion

### 3.1. Phase Morphology Analyses

**Figure 1:**
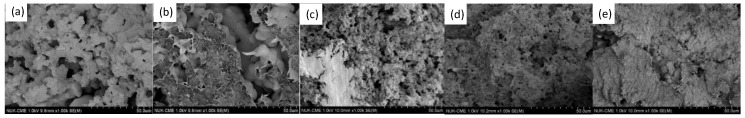
SEM images of phenoxy/PBT blends (1000 ×): (**a**) phenoxy/PBT = 1/9, (**b**) phenoxy/PBT = 3/7, (**c**) phenoxy/PBT = 5/5, (**d**) phenoxy/PBT = 7/3, and (**e**) phenoxy/PBT = 9/1.

In DSC traces, blends which possessed PBT contents resulted in a small enthalpic relaxation (unobvious T_g_s) in the blends. In the results of the thermal analyses, it was difficult to determine the miscibility of the blends based on the number of T_g_s. The miscibility of the phenoxy/PBT blends was directly observed by phase morphology in the study. The morphology of fracture surfaces of the phenoxy/PBT blends was examined using SEM. Figure 1a–e shows the cross-sections of the same blends with various compositions. The micrographs show particulate domains (5–10 μm) scattered across the fracture surfaces (cross-sections) of the blends for all compositions. From left to right, the micrographs show that for the PBT-rich blends, the PBT component (the aggregate particle domains) forms a continuous phase, with phenoxy forming a discrete phase. Similarly, for the phenoxy-rich blends, the phenoxy component forms a continuous phase, with the PBT component forming a discrete phase (the dispersed particulate domains). The SEM results demonstrate that the phase-separated morphology was observed in the as-blended phenoxy/PBT blends.

**Figure 2:**
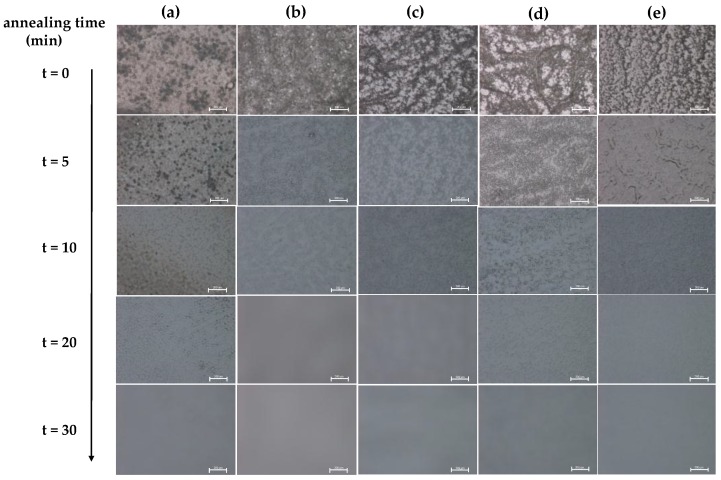
Optical micrographs of the phenoxy/PBT blends with various compositions annealed at 260 °C for various periods of time: (**a**) 9/1, (**b**) 7/3, (**c**) 5/5, (**d**) 3/7 and (**e**) 1/9.

To observe the effect of annealing on the phase morphology, the as-blended phenoxy/PBT blends were annealed at 260 °C for different periods of time and then examined using optical microscopy (OM). Figure 2a–e shows optical micrographs of the phenoxy/PBT blends with five compositions after being heated at 260 °C for various periods of time. Before annealing, the as-blended phenoxy/PBT blends of various compositions, including phenoxy-rich (dark domains) and PBT-rich (white domains), displayed phase separation. For the immiscible or partially miscible compositions, the phase domain sizes depended on the composition and decreased as the phenoxy weight fractions increased, as shown in the micrographs. According to the micrographs, in the immiscible blend, the size of the phase domain decreased, eventually disappearing as the annealing time increased. After annealing at 260 °C for 30 min, the blend’s morphology became homogeneous, suggesting that the specific interaction between phenoxy and PBT can improve miscibility in the phenoxy/PBT blend at this annealing temperature.

**Figure 3:**
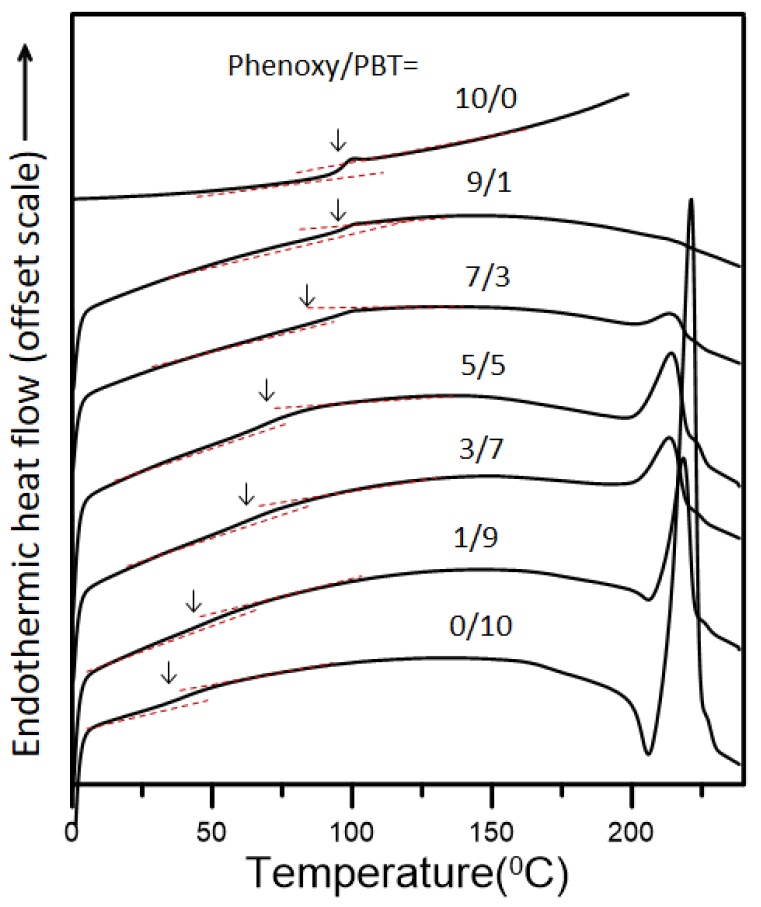
DSC traces of the phenoxy/PBT blends of various compositions annealed at 260 °C for 3 h.

Figure 3 shows DSC thermograms of the phenoxy/PBT blends of various compositions annealed at 260 °C for 3 h. The *T*_g_s for neat phenoxy and PBT were 95 °C and 36 °C, respectively. In the thermal analysis of polymer blends, a single *T*_g_ was used to evaluate the compatibilization of the phenoxy/PBT blends [2,3,26]. Figure 3 shows only a single, composition-dependent *T*_g_ for each blend, suggesting that phenoxy can blend with PBT to form a compatible or miscible blend. The enthalpic relaxation of semi-crystalline PBT was found to be considerably lower than that of its fully amorphous sample. In the study, blends which possessed higher PBT contents (with higher crystallization) resulted in unobvious T_g_s in the PBT rich blends [27]. As shown in Figure 2, an OM was used to examine the phenoxy/PBT blends, and the blends of various compositions annealed at 260 °C for 30 min were found to be transparent with no perceptible phase domains. When the annealing time was above 30 min, the phases of all the phenoxy/PBT blends, as revealed by OM, were domain-free and transparent. The OM examinations also showed a single-phase morphology for the heated blends of phenoxy with PBT. DSC and OM indicated that the blends were all miscible, with close mixing of the two polymeric segments of BT and the hydroxy ether of bisphenol-A in the heated blends.

**Figure 4:**
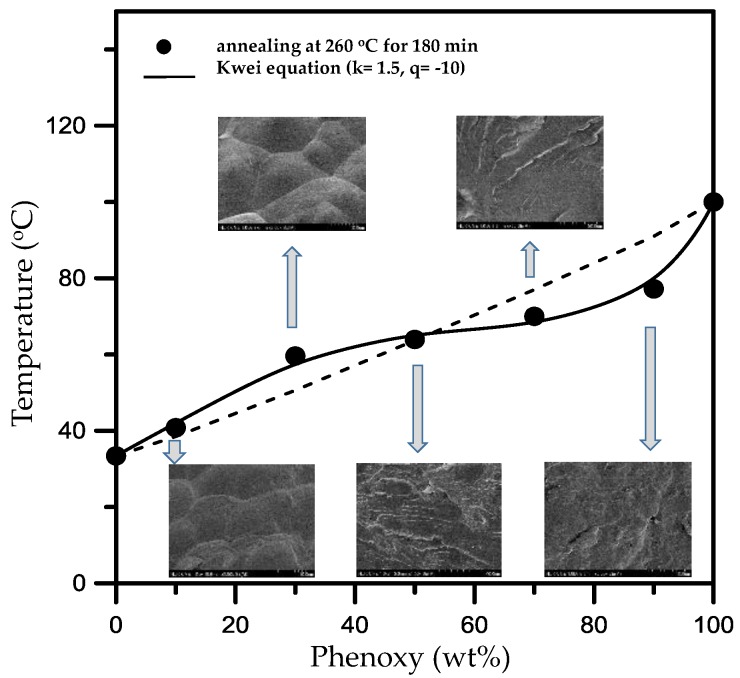
Plots of glass transition temperatures with SEM morphology versus compositions after isothermal treatment at 260 °C for 3 h.

The effect of high-temperature annealing on the phenoxy/PBT blends was also investigated by analyzing the *T*_g_ versus composition plot shown in Figure 4. Additionally, the insert blocks show the relevant SEM morphology of the annealed blends. The *T*_g_ was acquired from phenoxy/PBT blends that were post-annealed at 260 °C for 3 h, where the blends were homogenized into one phase, forming single *T*_g_ blends. The morphology and the *T*_g_–composition relationship clearly show that the annealed phenoxy/PBT blends are single-phase. Kwei [27] suggested an equation to describe the sigmoidal *T*_g_–composition curve:

(1) $$T_{g}=\frac{w_{1}T_{g1}+w_{2}kT_{g2}}{w_{1}+kw_{2}}+qw_{1}w_{2}$$

**Figure 5:**
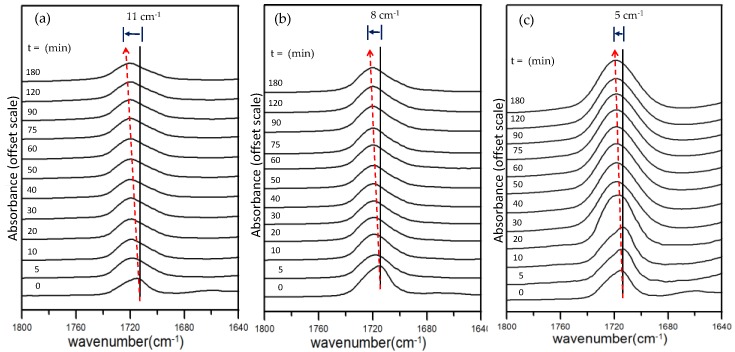
FTIR spectra in the carbonyl-stretching region of phenoxy/PBT blends after heating at 260 °C for various periods of time as indicated: phenoxy/PBT = (**a**) 9/1, (**b**) 5/5, and (**c**) 1/9.

In this equation, *w*_i_ is the weight fraction of component *i* and *T*_g_*_i_* is its glass transition temperature of component *i*, *k* represents the ratio of the thermal expansion coefficients or specific heat of phenoxy and PBT, and *q* is an empirical parameter related to the interchain interaction strength. Figure 5 shows the fitting of the *T*_g_ data to this relationship. The values were found to be 1.5 and −10 for *k* and *q* (at 260 °C for 180 min), respectively. The negative *q* value for the blends suggests stronger interactions between the components after annealing [3]. The above modeling equation for a well-mixed or homogeneous state can be used to describe the *T*_g_–composition relationship in phenoxy/PBT blends, indicating the formation of a miscible state with no inhomogeneous domain or phase separation. If the *T*_g_ elevation in the blends is due to intermolecular links through chemical reactions between the phenoxy and PBT, the maximum extent of reactions will occur in blends of phenoxy with PBT contents between 0 and 50 wt.%, as judged by the maximum increase in *T*_g_ within this composition range. Clearly, the extent of the chemical reactions between phenoxy and PBT were affected by not only the treatment temperature but also the blend compositions.

These results all suggest that the chemical reactions were induced by heat treatment of the phenoxy/PBT blend. An earlier study noted that favorable interchange reactions may proceed via the hydroxyl group (–OH) and the carbonyl groups (C=O) in phenoxy and aliphatic/aromatic polyester blends [28,29]. The C=O of polyester possess a positive charge on the carbon and a negative charge on the oxygen. The carbonyl carbon atom becomes electrophilic and thus reacts with nucleophiles in the group. Electrophilic compounds (Lewis bases) that react with the electron-poor (δ^+^) end of C=O are nucleophiles. Phenoxy, with the pendant hydroxyl group (electron-pair donor) in the repeating unit, enables interaction with the carbonyl group of PBT (proton-accepting functional group) in the polymer blends upon heating. Chemical interactions were observed between phenoxy and PBT upon annealing, which enhanced the miscibility and increased the *T*_g_ values of the phenoxy/PBT blends. Consequently, FTIR spectroscopy was used to probe the species that might participate in reactions.

### 3.2. Chemical Interactions in the Reactive Blend

Figure 5a–c shows the carbonyl absorption peaks in the IR spectra of the solution-cast phenoxy/PBT blends with 10, 50, and 90 wt.% of PBT, respectively, after heating at 260 °C for various periods of time. In each diagram, the carbonyl-stretching peak shifts to gradually higher frequencies as the heating time increases. For example, in diagram (a), the carbonyl absorption peak for the phenoxy/PBT blend (10 wt.% PBT) is seen to shift to a higher wavenumber by up to 11 cm^−1^ for the blend sample heated for 180 min at 260 °C. Diagram (b) shows a similar phenomenon for the carbonyl peak of the blend with 50 wt.% PBT. The shifts remain significant but are slightly reduced compared to the blend with 10 wt.% after heating for the same time at 260 °C. Note that the heating-induced shift in the carbonyl absorbance becomes gradually less apparent for the phenoxy/PBT blends with higher PBT contents of 50 and 90 wt.%. Diagram (c) shows that the phenoxy/PBT blend with 90 wt.% PBT exhibits a peak shift of only 5 cm^−l^ after heating for 180 min at 260 °C. This suggests that the chemical interaction was limited to a lesser extent when the PBT content was high in the blends and phenoxy became the limiting species.

One of our earlier studies illustrated that a transreaction arises in blends of phenoxy and copolyesters. Phenoxy, with a -OH in the structure, causes interaction with the C=O of copolyesters in polymer blends [3,4,5]. These chemical interactions result in the replacement of covalent bonds in the polymers, changing the linkages adjacent to the carbonyl group of polyesters. Polymers with various linkages adjacent to the carbonyl group exhibit a shift in the carbonyl IR absorption. C=O absorption bands at 1733, 1724, and 1713 cm^−1^ were assigned to carbonyl group stretching of aromatic-(CO)-O-aromatic (Ar-(CO)-O-Ar), aliphatic-(CO)-O-aliphatic (Al-(CO)-O-Al), and aromatic-(CO)-O-aliphatic (Ar-(CO)-O-Al) structures, respectively. In phenoxy/PBT blends, the C=O in the unreacted PBT chain is linked to aromatic and aliphatic linkages (Ar-(CO)-O-Al). However, exchange reactions change the linkages (two aliphatic) adjacent to the carbonyl group (Al-(CO)-O-Al).

**Figure 6:**
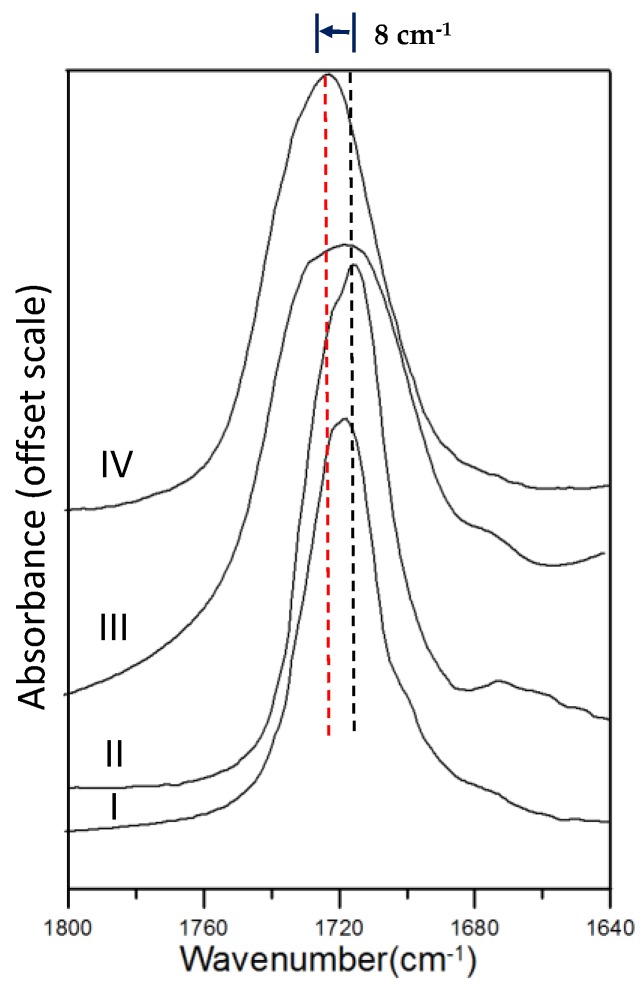
FTIR spectra of phenoxy/PBT (5/5) in the carbonyl group absorption region for blends annealing at 260 °C for 3 h. Spectrum (**I**) is the as-blended, (**II**) is the extracted solution, (**III**) is the heat-annealed blend, and (**IV**) is the leached solid.

Figure 6 shows carbonyl-stretching bands that from the phenoxy/PBT (5/5) blend annealed at 260 °C for 180 min. The figure shows the carbonyl absorbance shifts in the IR spectra of the heated blends (curve III) and leached solid (curve IV), along with the spectra of the as-blended sample and extracted solute, which are shown on the same diagram as curves I and II, respectively. In curve II, the carbonyl absorbance band of the heated extracted solute is similar to that of the as-blended sample, which suggests that the linkages adjacent to the carbonyl group of PBT (Ar-(CO)-O-Al) are the same for the as-blended sample and the extracted solute. In curve III, not only is the absorption peak of the carbonyl group at 1713 cm^−1^, but a new carbonyl absorbance band of Al-(CO)-O-Al at 1724 cm^−1^ is also observed. These suggest that two types of linkages adjacent to the carbonyl group (Al-(CO)-O-Al and Ar-(CO)-O-Al structures) are found in the heated blends. In curve IV, the carbonyl absorbance band shifts to a higher frequency for the leached solid samples, which suggests that extensive aliphatic linkages adjacent to the carbonyl group (Al-(CO)-O-Al) are formed in the leached solid samples. However, the leached solid sample primarily contains species of the exchange reaction of polymer segments with Al-CO)-O-Al and Al-(CO)-O-Ar structures. In the heat-annealed phenoxy/PBT blends, extensive exchange reactions produced a cross-linked network that formed residual solids in HFIP.

**Scheme 1:**
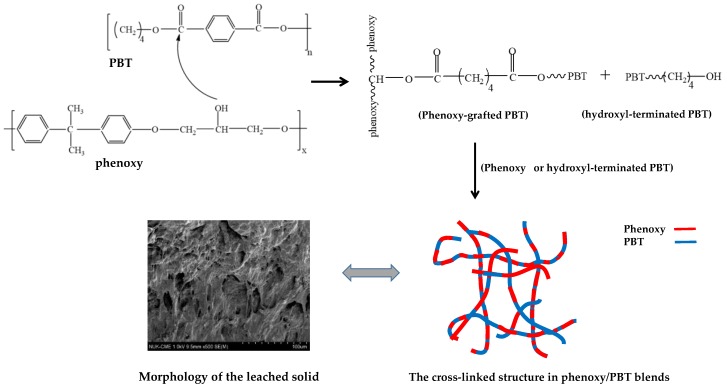
Exchange reactions in the annealed phenoxy/PBT blends.

The pendant hydroxyl group in phenoxy exhibits proton-donor characteristics when it interacts with carbonyl groups in PBT, which contains proton acceptors. Therefore, alcoholytic exchange can increase the compatibility of phenoxy with PBT in polymer blends upon annealing. Scheme 1 shows the mechanisms of the chemical interactions between the hydroxyl groups in phenoxy and the carbonyl groups in PBT. Polyesters react with a source of hydroxyl groups in the phenoxy in a phenoxy/PBT blend because –OH is a strongly activating group, Lewis base, or nucleophile, which attacks the δ^+^ end of the –O–C=O bond [25]. Clearly, in the heated blends, one product is fragmented PBT terminated with –OH, which originated from breakage of the carbonyl in joining with the proton in phenoxy. This can be viewed as alcoholysis. As proposed in the mechanisms, the reactions of the hydroxyl group of the phenoxy with an aliphatic/aromatic-linked carbonyl group of PBT initially led to the formation of a graft copolymer of phenoxy and PBT with an aliphatic/aliphatic carbonate link. Further reactions produced a complex mixture of polymer chain structures with an increasingly wider distribution of aromatic/aliphatic and aliphatic/aliphatic carbonate links. The degrees of reaction and the structures formed in the blends depended on the initial blend compositions and reaction conditions (i.e., heating temperatures and periods of time). Additionally, the progressive alcoholysis reaction results in the transformation of initial homopolymers into block copolymers and finally into random copolymers. The formed copolymers influence the compatibility of phenoxy and PBT. With extended heating, a highly linked structure was formed, which helped ensure blend miscibility. Note that the extensive alcoholysis reaction in the phenoxy/PBT blend formed a cross-linked network that was insoluble in the solvent and remained as a residual solid. Finally, the interlinked network elevated the glass transition temperatures of the blends upon heating. NMR was performed on blend samples before and after they were heated to 260 °C, but the similarity of the bonds made obtaining straight results difficult.

**Figure 7:**
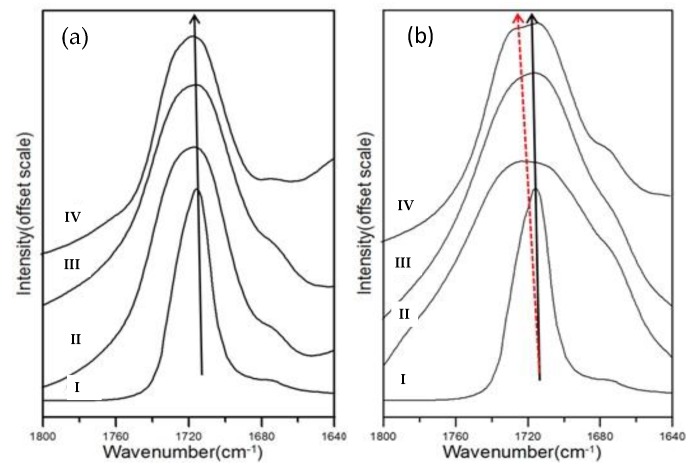
FTIR spectra of phenoxy/PBT (5/5) in the carbonyl absorption region for blends annealing at 260 °C for various periods of time. (**a)** extracted solute and (**b**) eached solids. In each diagram, spectrum (I) 0, (II) 30, (III) 60, and (IV) 90 min, rspectively.

Figure 7a shows the spectra for the Ar-(CO)-O-Al and Al-(CO)-O-Al (small amount) absorbance peaks for the species in the extracted solutes collected together and directly compared. Figure 7b shows the comparison spectra for the species in the leached solids. From the mechanisms, the crosslinked blend can be expected to have a higher extent of transreactions and alcoholysis exchanges than the extracted solutes that contain primarily linear molecules, fragmented PBT, or oligomeric phenoxy-PBT species with a Ar-(CO)-O-Al structure. This is apparent from the fact that the carbonyl stretching absorbance of the leached solid (i.e., crosslinked network) has a greater average shift of 10.5 cm^−1^ more than the solute portion (composed of unreacted components or fragmented PBT chains).

**Figure 8:**
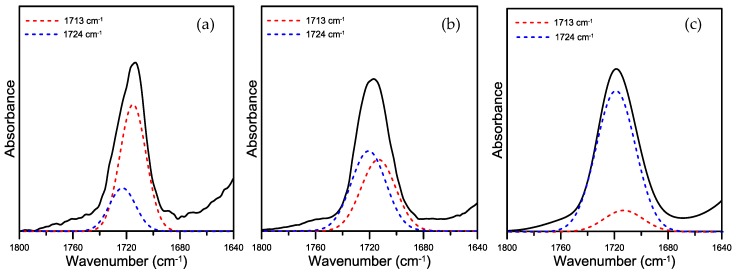
Deconvoluted IR absorbance peaks in the carbonyl-stretching region for phenoxy/PBT (9/1) after isothermal treatment at 260 °C for various periods of time: (**a**) 5 min, (**b**) 20 min, and (**c**) 90 min.

**Figure 9:**
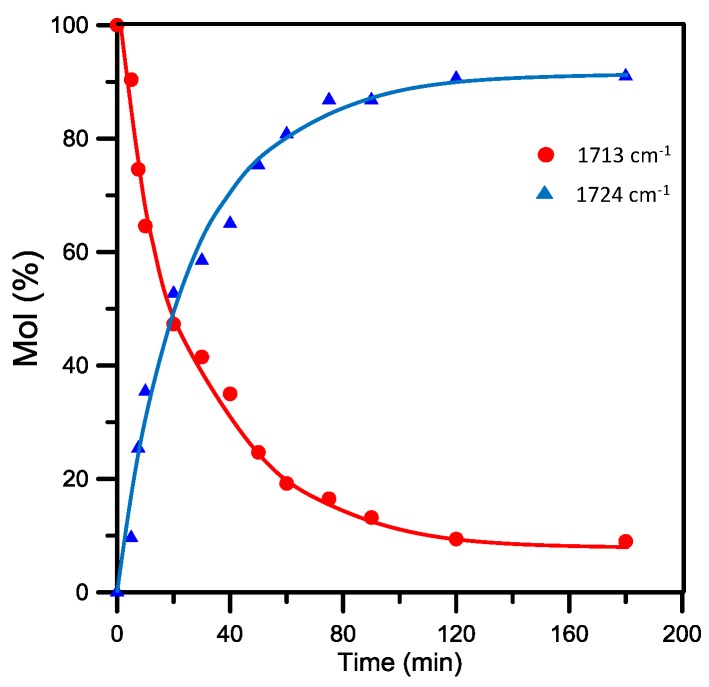
Mole fraction of specimens vs. heating time for phenoxy/PBT (9/1) at 260 °C.

The carbonyl-stretching band clearly shifted to the high-frequency side of the PBT carbonyl band, which strongly looks like that observed for the heated phenoxy/PBT blends. It would be reasonable to assign this band to the aliphatic/aliphatic carbonyl chain links of PBT. If we curve-resolve the spectrum showing the carbonyl-stretching band of the phenoxy/PBT (9/1) blend annealed for various periods of time into two components (aliphatic/aromatic and aliphatic/aliphatic-linked carbonyl groups), we obtain the results shown in Figure 8. The shift in the C=O IR absorption of the annealed phenoxy/PBT blend was investigated using the commercial software “PeakFit 4.12” (V 12.0, Systat Software Inc., San Jose, CA, USA) to observe the composition of the Ar-(CO)-O-Al and Al-(CO)-O-Al species in the PBT sequence. In the annealed phenoxy/PBT blend, the carbonyl-stretching band was fitted to the carbonyl absorption bands at 1713 and 1724 cm^−1^, assigned to carbonyl-stretching of the Al-(CO)-O-Al and Ar-(CO)-O-Al structures, respectively. Assuming that the absorption coefficients of the two C=O bands assigned to the aliphatic/aliphatic and aromatic/aliphatic carbonyl groups are similar in magnitude, we can calculate the fraction of each in the phenoxy/PBT blend as a function of the annealing time at 260 °C. Figure 9 shows the mole fractions of the aromatic/aliphatic (1713 cm^−1^) and aliphatic/aliphatic (1724 cm^−1^)-linked carbonyl groups in the phenoxy/PBT (9/1) blend annealed at 260 °C for various periods of time. As the alcoholysis reaction in the phenoxy/PBT blend progressed, the aliphatic/aliphatic linked carbonyl groups increased with the annealing time. Conversely, the aromatic/aliphatic-linked carbonyl groups decreased as the annealing time increased. The original aromatic/aliphatic-linked carbonate in the linear PBT chains formed aliphatic/aliphatic carbonyl ester links between phenoxy and PBT for the phenoxy/PBT blend annealed at 260 °C for 3 h. Having established spectroscopically that no further reaction happened at 260 °C in the blend, we believe that we observed the effect of an incomplete reaction due to cross-linking and gelation. Phenoxy-rich blends were observed to gel after 3 h at 260 °C and were no longer completely soluble in solvent.

### 3.3. Effect of Chemical Interactions on Crystallization Behavior

**Figure 10:**
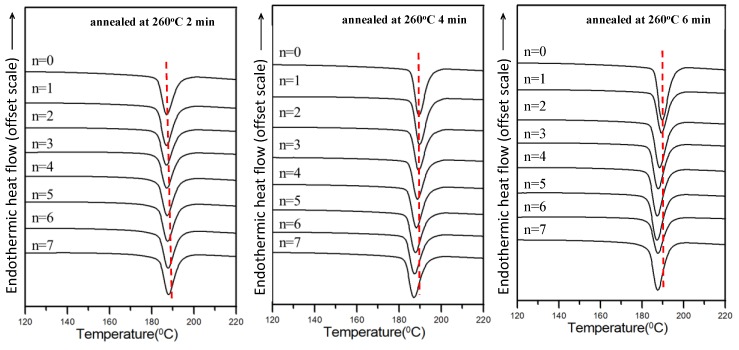
DSC traces of of phenoxy/PBT = 1/9 samples after cycles of annealing at 260 °C for various periods of time followed by cooling.

In reactive blends, the progressive exchange reaction results in the transformation of the initial homopolymers into block copolymers and finally into random copolymers. The formed copolymers influence the miscibility of the blends. Exchange reactions also influence the crystallization of semicrystalline polymers in the blends. The transition to block or random copolymers decreases the crystallinity because of the dissimilarity of the copolymer chemical units [23,30]. In this work, the effect of the progressive exchange reaction on the crystallization of PBT was investigated for the heated phenoxy/PBT blends. As PBT-rich blends have crystalline characteristics, the nonisothermal crystallization of phenoxy/PBT = 1/9 blends was used to study this effect. Figure 10 shows DSC thermograms of the nonisothermal crystallization behavior of phenoxy/PBT = 1/9 blends that experienced seven cycles of annealing between 0 °C and 260 °C. The crystallization temperature (*T*_c_) of PBT decreased as the annealing time increased and/or the number of cycles of annealing to 260 °C increased. This suggests that the alcoholysis reaction between phenoxy and PBT takes place not only in the melted sample, but also in the amorphous phase of the sample under thermal annealing below the melting temperature. Increasing the annealing time and the number of cycles of annealing increases the alcoholytic exchange between phenoxy and PBT, and therefore copolymers are observed in the blends.

**Figure 11:**
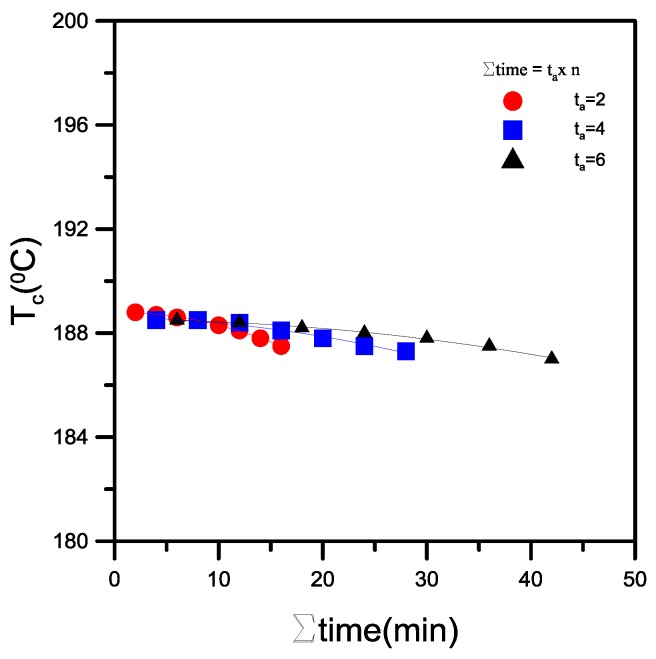
Effect of the Σ*t*_a_ on *T*_c_ for the phenoxy/PBT = 1/9 sample.

Figure 11 shows *T*_c_ as a function of the total annealing time for the phenoxy/PBT = 1/9 sample. In this experiment, the annealing time varied from 0 to 6 min. The *T*_c_ decreased faster when n was higher for a given total annealing time. These results suggest that the alcoholysis reaction intensified as the total annealing time increased, resulting in a stepwise increase in the copolymer content. In the blends, the digression of *T*_c_ upon annealing increased with the annealing time, owing to the inhibition of crystallization by the shortening of the PBT segments with Ar-(CO)-O-Al carbonyl groups in the copolymers. In the crystalline polymer, the basic units of the crystalline polymer morphology included crystalline lamellae consisting of arrays of folded chains [31]. The decrease in the degree of crystallization of PBT as the total annealing time (Σ*t*_a_) increased reveals the reduction of regular crystalline lamellae in PBT. The behavior that was expected as a consequence of the copolymers increased upon annealing because of the inhibition of crystallization by alcoholytic exchange.

## 4. Conclusions

As-blended phenoxy/PBT blends exhibited immiscible phases, and the initially phase-separated blends eventually merged into a homogeneous phase with a single *T*_g_ on annealing at 260 °C for 180 min. The presence of hydroxyl and carbonyl groups in the constituent polymers does not guarantee hydrogen bonding, which would induce miscibility. However, chemical exchange reactions upon annealing probably caused phase homogenization in the phenoxy/PBT blends. The results of this study demonstrate that heat annealing can induce phase homogenization in phenoxy/PBT blends, which exhibit chemical interaction-induced miscibility. FTIR analysis revealed heat-induced shifting of the carbonyl-stretching band due to changes in the linkages adjacent to the carbonyl group of PBT. In this work, the pendant hydroxyl group in phenoxy exhibited proton-donor characteristics when it interacted with carbonyl groups in PBT that contain proton acceptors. In the phenoxy/PBT blend, the carbonyl group in the original unreacted PBT chain was linked to aromatic and aliphatic linkages (Ar-(CO)-O-Al). However, alcoholysis changed the linkages adjacent to the carbonyl group (Al-(CO)-O-Al). The reactions of the hydroxyl group of phenoxy with an aliphatic/aromatic-linked carbonyl group of PBT initially led to the formation of a graft copolymer of phenoxy and PBT with an aliphatic/aliphatic carbonyl link. Further reaction produced a complex mixture of polymer chain structures with an increasingly wider distribution of aromatic/aliphatic and aliphatic/aliphatic carbonate links. Additionally, the progressive alcoholysis reaction changed the initial homopolymers into block copolymers and finally into random copolymers. The formed copolymers influenced the compatibility of phenoxy and PBT. Therefore, alcoholytic exchange can increase the compatibility of phenoxy with PBT in polymer blends upon annealing. The chemical interactions in the phenoxy/PBT blends affected not only the compatibility of the blends but also the crystallization behavior of the semicrystalline polymer in the blends. In the reactive amorphous/semicrystalline polymer blends, arrays of folded chains in the crystalline lamellae of PBT were interrupted by the copolymers formed in the exchange reaction. These reduced the ordering of the polymer chain, subsequently reducing the degree of crystallization of PBT in the blends.
